# Persistent SARS-CoV-2 infection in SCID rats reflects immune vulnerability of the upper airway and trachea

**DOI:** 10.1128/spectrum.04105-25

**Published:** 2026-10-01

**Authors:** Kaya Miyazaki, Nozomi Shiwa-Sudo, Naoko Iwata-Yoshikawa, Yusuke Sakai, Kazuto Yoshimi, Tomoji Mashimo, Tadaki Suzuki, Eiichi N. Kodama, Hideki Hasegawa, Noriyo Nagata

**Affiliations:** 1 Graduate School of Medicine, Tohoku University 13101, Sendai, Japan; 2 National Institute of Infectious Diseases, Japan Institute for Health Security 119813, Tokyo, Japan; 3 Division of Animal Genetics, Laboratory Animal Research Center, Institute of Medical Science, The University of Tokyo 26430, Tokyo, Japan; 4 Tohoku Medical Megabank Organization, Tohoku University 13101, Sendai, Japan; 5 Internatinal Research Institute of Disaster Science, Tohoku University 13101, Sendai, Japan; 6 Institute for Vaccine Research and Development, Hokkaido University 12810, Sapporo, Japan; University of Georgia, Athens, Georgia, USA

**Keywords:** SARS-CoV-2, animal models, persistent infection, SCID, rats

## Abstract

In immunocompromised patients, persistent severe acute respiratory syndrome coronavirus 2 (SARS-CoV-2) infection carries the risk of prolonged viral shedding and emergence of new variants. To reproduce the key features of persistent SARS-CoV-2 infection and evaluate its transmissibility, mouse-passaged SARS-CoV-2 was administered intranasally to severe combined immunodeficiency (SCID) rats deficient in *Rag2* and *Il2rg* genes. The virus replicated in SCID rats for at least 6 weeks without causing severe symptoms. The main replication sites were the nasal cavity and trachea, and replicating viruses were barely detectable in the lungs. Infected areas within the nasal cavity and trachea showed minimal infiltration of neutrophils and M2 macrophages; however, a strong inflammatory response was observed in the lungs. Serial sequencing of the viral genome revealed accumulation of persistent mutations, predominantly in regions encoding nonstructural proteins. The persistently shed virus retained its infectivity, enabling its transmission to uninfected SCID rats. This model provides new insights into the immunological and tissue-associated features of persistent SARS-CoV-2 infection and offers a practical research platform for developing therapeutic interventions.

**IMPORTANCE:**

Persistent severe acute respiratory syndrome coronavirus 2 (SARS-CoV-2) infection in immunocompromised individuals promotes viral evolution and ongoing transmission, yet mechanistic studies have been limited by the absence of appropriate animal models. Existing mouse systems do not support longitudinal sampling or evaluate transmission, leaving critical questions unanswered. We established an SCID rat model that enables repeated noninvasive sampling from the same individuals, allowing longitudinal analysis of viral dynamics during prolonged infection. This approach revealed the emergence, disappearance, and fixation of viral mutations over time. Persistently infected rats also retained infectious and transmitted virus to naïve SCID rats, providing experimental evidence that prolonged infection preserves the transmission potential in this model. Persistent viral replication localized to the nasal cavity and trachea may be relevant to the upper-airway persistence reported in some immunocompromised patients. By capturing selected features of persistent SARS-CoV-2 infection under profoundly immunodeficient conditions, this model provides a valuable platform for investigating viral persistence, evolution, and transmission.

## INTRODUCTION

Severe acute respiratory syndrome coronavirus 2 (SARS-CoV-2) variants continue to pose a threat to global health, particularly in vulnerable populations, such as immunocompromised individuals (1, 2). Patients with underlying conditions, such as hematologic malignancies, organ transplants, primary immunodeficiencies, or HIV infection, face an increased risk of adverse coronavirus disease 2019 (COVID-19) outcomes and are more likely to experience prolonged infection (3–6). In such hosts, SARS-CoV-2 can persist for months or even over a year, during which the virus accumulates mutations (7–14). These persistent infections, which promote intrahost viral evolution, have drawn increasing attention as potential sources of novel variants that retain both infectivity and transmissibility (15–19).

Various animal models have contributed significantly to our understanding of SARS-CoV-2 pathogenesis, transmission, and immunity (20–22). However, in commonly used models, such as K18-hACE2 transgenic mice, Syrian hamsters, and ferrets, infection typically resolves within 1–2 weeks and fails to recapitulate the prolonged persistence and diverse evolutionary dynamic characteristics of immunodeficient patients. Although mouse models of persistent infection have elucidated tissue-specific immunity and the efficacy of antiviral therapeutics in immunocompromised hosts (23, 24), their small body size limits longitudinal sampling of the upper respiratory tract, hindering the continuous tracking of viral evolutionary dynamics within the same animal. Furthermore, the transmissibility of persistently infected hosts has not yet been comprehensively evaluated in small animal models.

To address these limitations, we used a rat model. Although wild-type (WT) rats are only partially susceptible to SARS-CoV-2 infection (25), their larger body size allows for repeated sampling and detailed longitudinal analyses (26–28). Rats also possess physiological characteristics that allow stable observation over an extended period (29), and well-established assessment systems, such as respiratory function monitoring and pharmacokinetic analysis, are available (28, 30).

Therefore, we hypothesized that immunodeficient rats infected with mouse-passaged SARS-CoV-2 could overcome the limitations of prior susceptibility models and establish a persistent infection similar to that observed in immunocompromised patients. To test this, we utilized a severe combined immunodeficiency (SCID) rat model that was deficient in both the *Rag2* and *Il2rg* genes and intranasally infected with a mouse-passaged SARS-CoV-2 strain, QHmusX (31). QHmusX, derived from a B-lineage virus, carries a Q498H mutation in the spike receptor-binding domain, which enhances its binding affinity to both mouse and rat ACE2 (31, 32). This SCID rat model also lacks functional B, T, and NK cells while maintaining physiological stability conducive to long-term monitoring (33).

Taken together, these characteristics enable the analysis of tissue-specific determinants of viral persistence and clearance, as well as the longitudinal dynamics of intra-host viral evolution and transmission potential of persistently infected hosts and provide new insights into the pathogenesis of persistent SARS-CoV-2 infection in immunodeficient hosts.

## RESULTS

### Establishment of a persistent SARS-CoV-2 infection in SCID rats

**Fig 1:**
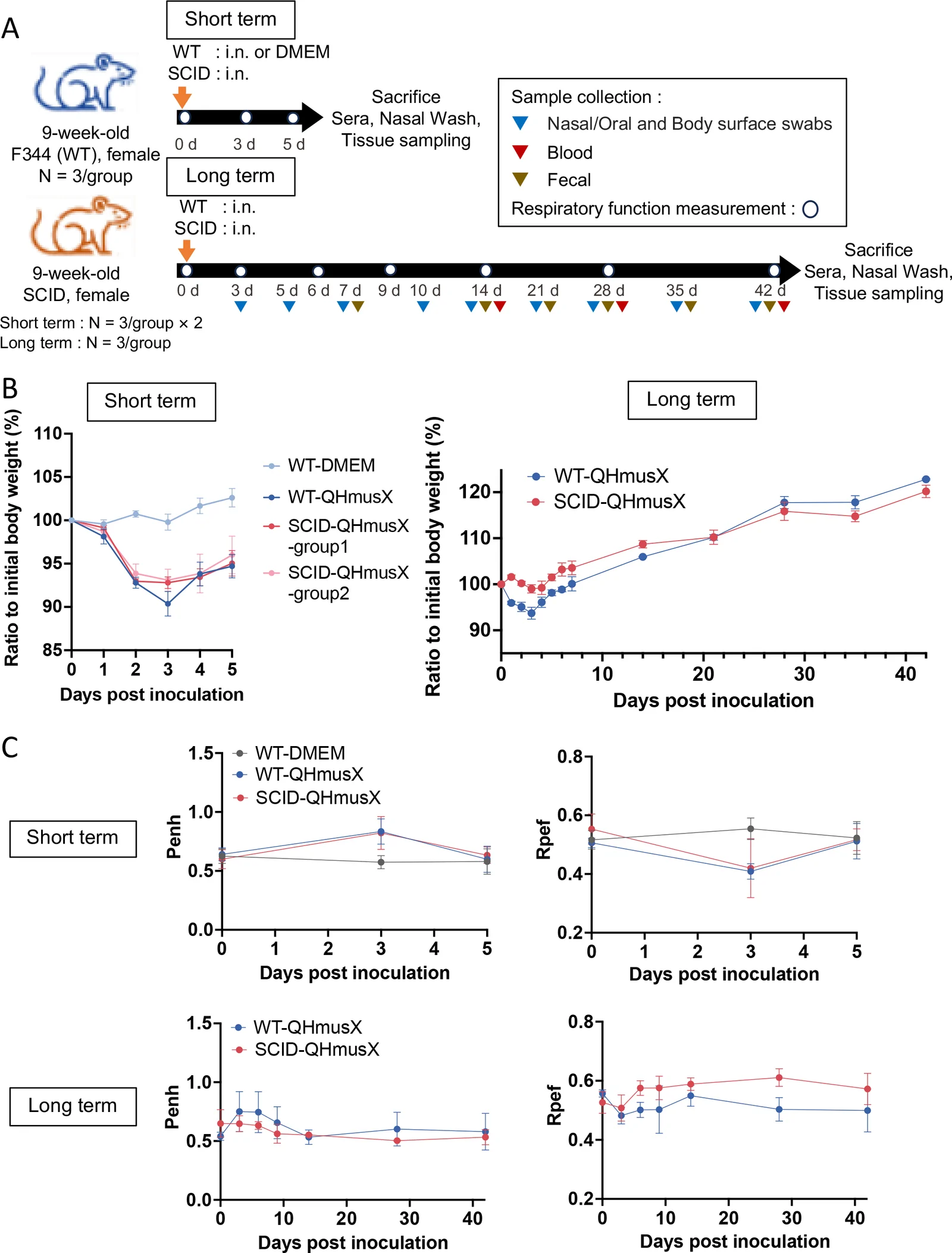
Experimental design and clinical outcomes of SCID and WT F344/Jcl rats following infection with SARS-CoV-2. (**A**) Experimental design. F344-*Il2rg/Rag2^em1Iexas^* (referred to as SCID) and F344/Jcl (referred to as WT) rats (female, 9 weeks old) were inoculated intranasally with 10^5^ TCID_50_ of mouse-passaged SARS-CoV-2 (QHmusX strain) or vehicle control (DMEM). Rats were monitored over the short (5 days, two independent groups of SCID rats, one group of WT rats, *n* = 3) or long term (42 days, one group of each rat strain, *n* = 3). Time points for sample collection are designated as follows: blue triangles, nasal/oral and body surface swabs; and red triangles, blood; brown triangles, feces. Respiratory function was assessed by whole-body plethysmography (circles). (**B**) Changes in body weight. The percentage change in body weight (relative to baseline) was measured over the short (left) and long (right) terms. Both SCID and WT rats showed transient weight loss, but all recovered subsequently. Data are presented as the mean ± SEM. (**C**) Respiratory function parameters. Time course of enhanced pause (Penh) and time to peak expiratory flow ratio (Rpef) as measured by whole-body plethysmography over the short- (upper panels) and long-term (lower panels) study. Data are presented as the mean ± SEM.

To investigate persistent SARS-CoV-2 infection in immunodeficient hosts, we first established an experimental model based on SCID rats lacking the *Rag2* and *Il2rg* genes (F344-*Il2rg/Rag2^em1Iexas^*). WT F344 rats were used as the controls (Fig. 1A). Female rats (9 weeks old; *n* = 3/group) from both strains were inoculated intranasally with 10^5^ TCID_50_ of the mouse-passaged SARS-CoV-2 strain QHmusX. For the short-term experiment, two independent SCID cohorts (groups 1 and 2; Day 5 p.i.; *n* = 3 per cohort) and one WT cohort (Day 5 p.i.; *n* = 3) were analyzed. Two independent SCID cohorts were included to assess the consistency of early infection findings. Both cohorts underwent identical inoculation, sampling, and analytical procedures. Independent long-term SCID and WT cohorts were analyzed separately on Day 42 p.i. (*n* = 3 per cohort).

Following infection, both SCID and WT rats exhibited a transient loss of body weight, although all rats recovered to their baseline levels (Fig. 1B). In both groups, respiratory function, as assessed by whole-body plethysmography, revealed temporary alterations in enhanced pause (Penh) and peak expiratory flow (Rpef) rates at 3 days p.i., which returned to baseline levels by Day 5 (Fig. 1C). No rat exhibited sustained respiratory dysfunction.

**Fig 2:**
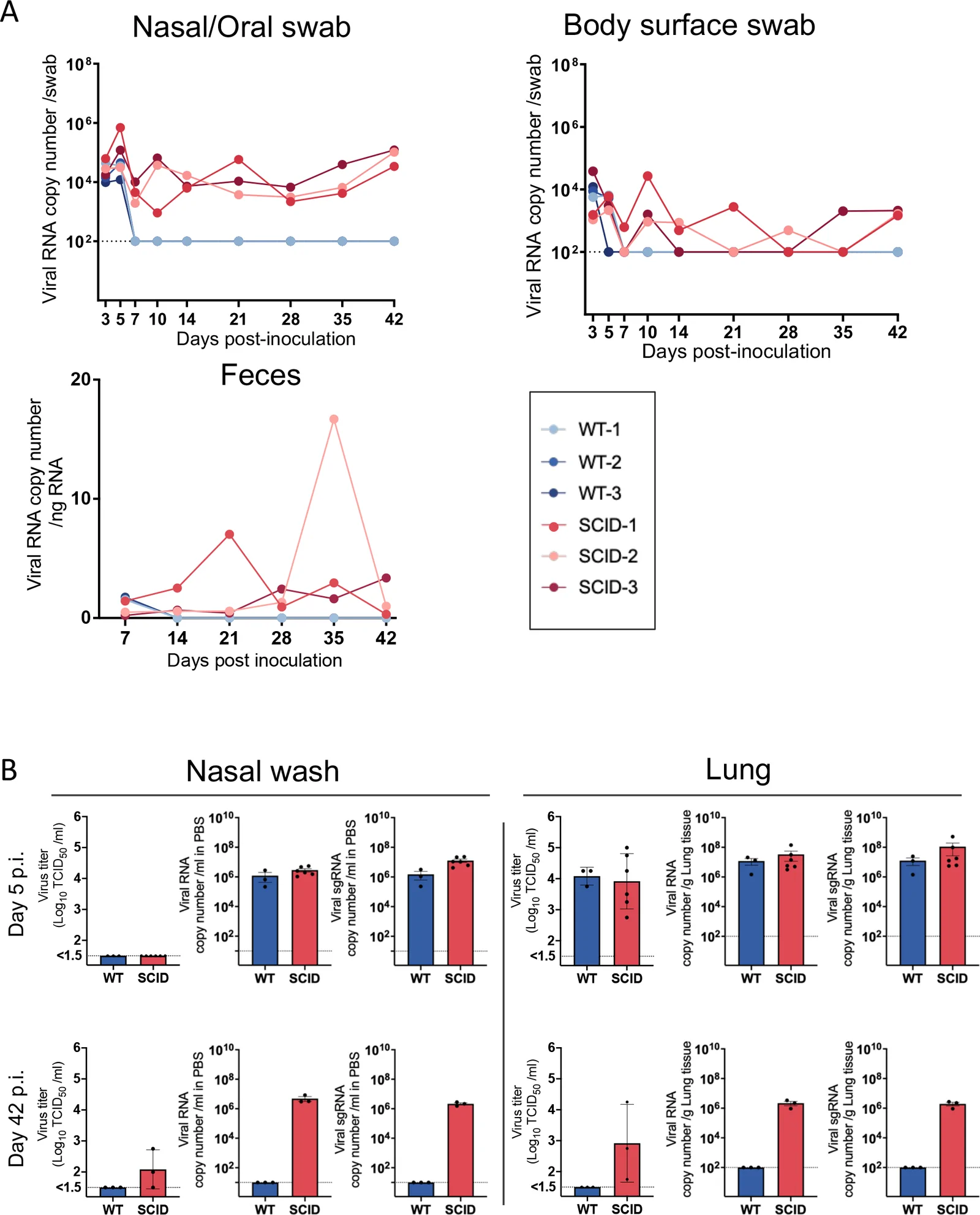
Persistent SARS-CoV-2 infection in SCID rats. (**A**) Detection of viral RNA in swab and fecal samples. Temporal changes in the viral RNA copy number in nasal/oral (upper left) and body surface (upper right) swabs from WT F344/Jcl (WT; blue shades) and SCID (red shades) rats. Each line represents an individual rat (#1–3 in each group). Dotted lines indicate the limit of detection. (**B**) Quantification of viral RNA in nasal wash and lung homogenates. The panels show the infectious virus titer, viral RNA copy number, and subgenomic RNA copy number (left to right) in nasal wash and lung homogenates from WT and SCID rats at days 5 (upper panels) and 42 (lower panels) p.i.. Dotted lines indicate the limit of detection.

Despite the similar clinical outcomes in both groups, there were marked differences in the patterns of viral clearance. In WT rats, viral RNA in nasal/oral swabs, body surface swabs, and fecal samples became undetectable by Day 7 p.i. In contrast, viral RNA isolated from SCID rats remained detectable in all samples until Day 42 p.i. (Fig. 2A). Low-level genomic viral RNA was detected in terminal blood samples from several SCID rats at Day 5 p.i. (Table 1) and in one longitudinal blood sample collected at Day 14 p.i. (126 copies/10 µL blood). However, genomic viral RNA was not detected in the blood of SCID rats on Day 42 p.i. Notably, subgenomic RNA was below the assay limit in all blood samples from both WT and SCID rats, whereas subgenomic RNA was detected in tissue specimens from SCID rats (Table 2). Infectious viruses and viral RNA were present in nasal washes and lung homogenates from SCID rats at both days 5 and 42 p.i., whereas all samples from WT rats were negative by Day 42 p.i. (Fig. 2B). SCID rats lacking B cells did not mount a humoral immune response, resulting in sustained viral replication, whereas WT rats developed robust neutralizing antibodies by Day 42 p.i. (Fig. S1).

**TABLE 1:** Distribution of viral RNA in rat tissues at 5 and 42 days post-inoculation^*a*^

Group	WT F344/Jcl rats			SCID rats								
Day of sacrifice	5 days p.i.			5 days p.i.						42 days p.i.		
Animal #	#1	#2	#3	#1	#2	#3	#4	#5	#6	#1	#2	#3
Sample												
Brain	–	–	–	–	–	–	–	–	–	–	–	–
Lung	99.08	264.40	130.96	15.70	48.32	212.96	10.86	6.42	51.70	325.03	36.35	47.95
Heart	0.25	0.17	–	–	–	–	–	–	–	–	–	–
Liver	–	–	–	–	4.96	–	–	–	–	–	–	–
Spleen	–	0.03	0.05	–	23.70	–	–	–	–	–	–	–
Kidney	–	–	–	–	–	–	–	–	–	–	–	–
Pancreas	–	–	–	–	–	–	–	–	–	–	–	–
Small intestine	0.26	0.01	0.01	NE	NE	NE	NE	NE	NE	0.05	0.23	0.25
Large intestine	0.01	0.02	0.03	–	–	–	–	–	–	–	–	0.06
Blood	–	–	–	0.4	0.72	0.13	–	–	–	–	–	–
Cervical lymph nodes	0.26	0.14	0.28	–	–	–	–	–	–	–	–	–

^*a*^ –, below the limit of detection; viral RNA copy number per ng of total RNA; NE, not examined; p.i., post-inoculation.

**TABLE 2:** Distribution of subgenomic viral RNA in rat tissues at 5 and 42 days post-inoculation^*a*^

Group	WT F344/Jcl rats			SCID rats								
Day of sacrifice	5 days p.i.			5 days p.i.						42 days p.i.		
Animal #	#1	#2	#3	#1	#2	#3	#4	#5	#6	#1	#2	#3
Sample												
Brain	–	–	–	–	–	–	–	–	–	–	–	–
Lung	1,353.80	2,461.63	401.74	195.47	757.68	3,932.25	57.22	57.06	324.82	752.67	365.74	212.00
Heart		5.02	–	–	–	–	–	–	–	–	–	–
Liver	–	–	–	–	–	–	–	–	–	–	4.53	3.56
Spleen	44.65	–	–	–	–	–	–	–	–	–	–	42.58
Kidney	–	–	–	–	–	–	–	–	–	–	–	–
Pancreas	–	–	–	–	–	–	–	–	–	–	–	–
Small intestine	3.54	–	–	NE	NE	NE	NE	NE	NE	2.79	4.94	3.26
Large intestine	–	–	–	–	–	–	–	–	–	–	–	–
Blood	–	–	–	–	–	–	–	–	–	–	–	–
Cervical lymph nodes	19.29	12.14	29.65	–	–	–	–	–	–	–	–	–

^*a*^ –, below the limit of detection; viral RNA copy number per ng of total RNA; NE, not examined; p.i., post-inoculation.

Quantitative analysis of the distribution of viral RNA across organs revealed that the highest viral load was present in the lung tissue, which persisted in SCID rats until Day 42 p.i. (Table 1). Subgenomic viral RNA, indicative of active viral replication, was consistently detected in multiple organs of the SCID rats throughout the observation period, confirming ongoing viral replication rather than the persistence of viral RNA (Table 2). In contrast, viral RNA was detected only transiently and at low levels in WT rats. Furthermore, in SCID rats, low levels of viral RNA were occasionally detected in highly vascularized organs, such as the spleen and liver, suggesting possible systemic dissemination during the early phase of infection. Also, viral RNA was intermittently detected, albeit at low abundance, in intestinal tissues from WT rats at Day 5 and SCID rats at Day 42, whereas subgenomic RNA was detected rarely from these tissues of either of these rats (Tables 1 and 2). Immunohistochemistry yielded no definitive antigen-positive findings in the intestinal mucosal epithelium in either group.

### Maintenance of virus antigen-positive cells in the upper respiratory tract and trachea

**Fig 3:**
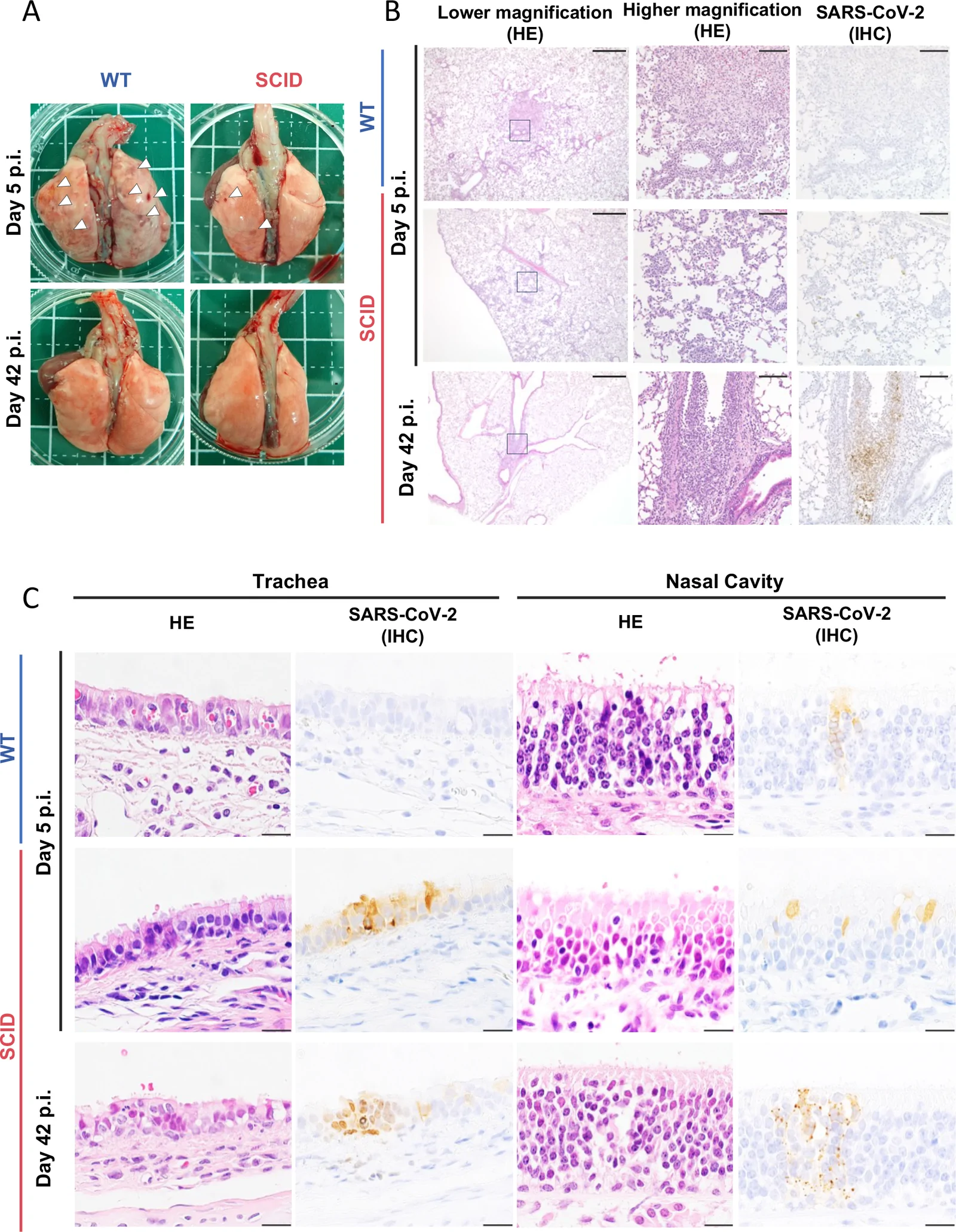
Histopathological comparison of respiratory tract tissues in wild-type and SCID rats following SARS-CoV-2 infection. (**A**) Gross pathology of the lungs. Lungs from WT F344/Jcl (left) and SCID rats (right) at 5 (upper panels) and 42 days (lower panels) p.i. with the QHmusX strain. White arrowheads indicate the lesion sites. Photo credit: Kaya Miyazaki, National Institute of Infectious Diseases, Japan Institute for Health Security. (**B**) Histopathological findings in the lungs. Lung tissues from WT (upper panels) and SCID rats (lower panels) were harvested at 5 and 42 days p.i.. Scale bar = 500 µm. Low-magnification (×40) H&E staining (first column), high-magnification (×200) H&E staining (second column), and SARS-CoV-2 antigen immunohistochemical staining (third column) are shown. Scale bar = 100 µm. (**C**) Histopathological findings in the trachea and nasal cavity. Trachea (left columns) and nasal cavity (right columns) tissues were harvested from WT (upper panels) and SCID rats (lower panels) at 5 and 42 days p.i.. H&E staining (left) and SARS-CoV-2 antigen immunohistochemical staining (right) are shown. Scale bar = 20 µm.

To elucidate the pathology underlying persistent infection by SARS-CoV-2, we examined respiratory tissues from WT and SCID rats at 5 and 42 days p.i. Gross pathological analysis of WT rats at Day 5 p.i. revealed mild but extensive pneumonic lesions, whereas SCID rats exhibited only a few localized lesions (Fig. 3A). By Day 42 p.i., the inflammatory lesions had resolved in both groups. Despite the persistence of the viral load in the SCID rats, we found no progressive, macroscopically observable lesions. Histological analysis of H&E-stained lung sections on Day 5 p.i. (Fig. 3B) revealed desquamated cells in the bronchioles and alveoli of WT rats, accompanied by a prominent infiltration of inflammatory cells (mainly neutrophils and macrophages). Viral antigen-positive cells were focally detected, predominantly around the bronchioles. In contrast, inflammatory cell infiltration in the same regions in the SCID rats was milder and more sporadic. Analysis of the respiratory tract tissues revealed distinct differences in viral localization between the two groups. At 5 d.p.i., viral antigens were barely detected in the tracheas of WT rats, with occasional positive cells in the nasal cavity. By 42 d.p.i., no viral antigen-positive cells were observed in WT rats (Fig. 3C; Fig. S2). Viral antigen-positive cells were detected in the nasal cavity of all SCID rats (six rats in total, consisting of two groups of three) at 5 d.p.i., in the trachea of 5/6 SCID rats (from two groups of three) at 5 d.p.i., and in the lungs of 5/6 SCID rats (from two groups of three) at 5 d.p.i. Antigen-positive cells persisted in the nasal cavity and trachea of all SCID rats in the 42 d.p.i. group (*n* = 3). In all SCID rats, the viral antigens localized consistently as small clusters within the epithelial layer of the tissues. Viral antigens were present in the lungs of two of the three SCID rats in this group, although these cells were confined to regions adjacent to the trachea or were observed only as single cells or small clusters within highly localized foci. To further characterize the types of infected cells and the immune microenvironment associated with this persistence, we performed multiplex immunofluorescence and spatial analyses.

### Cellular localization and immune cell infiltration during the early and persistent stages of infection

**Fig 4:**
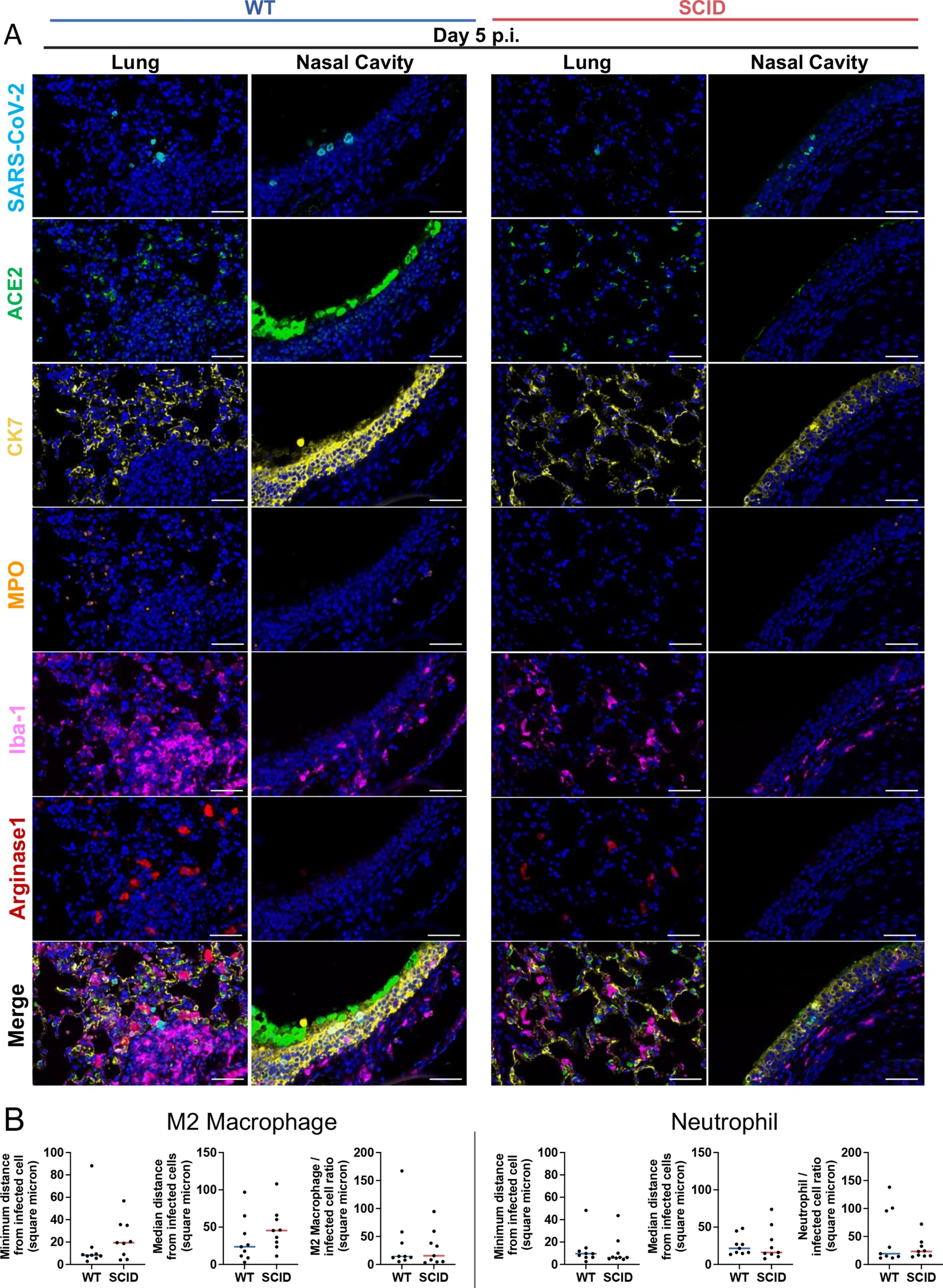
Multiplex immunofluorescence analysis of epithelial cell tropism and immune cell infiltration at 5 days post-infection. (**A**) Representative multiplex immunofluorescence-stained images of the lung and nasal cavity tissues from WT F344/Jcl rats and SCID rats at 5 dpi, showing SARS-CoV-2 antigen (cyan), ACE2 receptor (green), the epithelial cell marker CK7 (yellow), the neutrophil marker MPO (orange), the M2 macrophage marker arginase-1 (red), and the pan-macrophage marker Iba-1 (magenta). The bottom row shows merged images of all channels. The DAPI nuclear stain (blue) is also shown. Scale bar = 50 µm. (**B**) Quantitative analysis of immune cell infiltration. Quantification of minimum distance, median distance, and immune cell ratio per infected cell (Iba-1, Arginase-1,1^+^ M2 macrophages, left panels; and MPO,^+^ neutrophils, right panels) in the lung. Each data point represents measurements from three fields per individual (*n* = 3). WT (blue), SCID (red).

Multiplex immunofluorescence analysis conducted to identify receptor utilization and infected cells confirmed strong ACE2 expression in regions harboring SARS-CoV-2 antigens on Day 5 p.i. in both WT and SCID rats. At the infection site, antigen-positive cells colocalized with cytokeratin 7 (CK7), confirming their affinity for epithelial cells (Fig. 4A). Multiplex staining revealed that the number of Iba-1-positivee macrophages in the lungs of SCID rats was markedly lower than that in the lungs of WT rats, in which abundant infiltration was observed. Furthermore, Arg1-positive M2 macrophages in WT lungs tended to accumulate closer to infected cells. In contrast, both WT and SCID rats exhibited limited immune cell infiltration into the nasal mucosa. Based on these observations, we focused on M2 macrophages and neutrophils to analyze their spatial relationships with infected cells in the lungs. Quantitative spatial analysis of WT lungs confirmed that M2 macrophages were localized close to infected cells, whereas those in SCID rats were located at greater distances from the infected cells (Fig. 4B). Additionally, neutrophils were positioned close to the infected cells with a similar spatial distribution in both groups.

**Fig 5:**
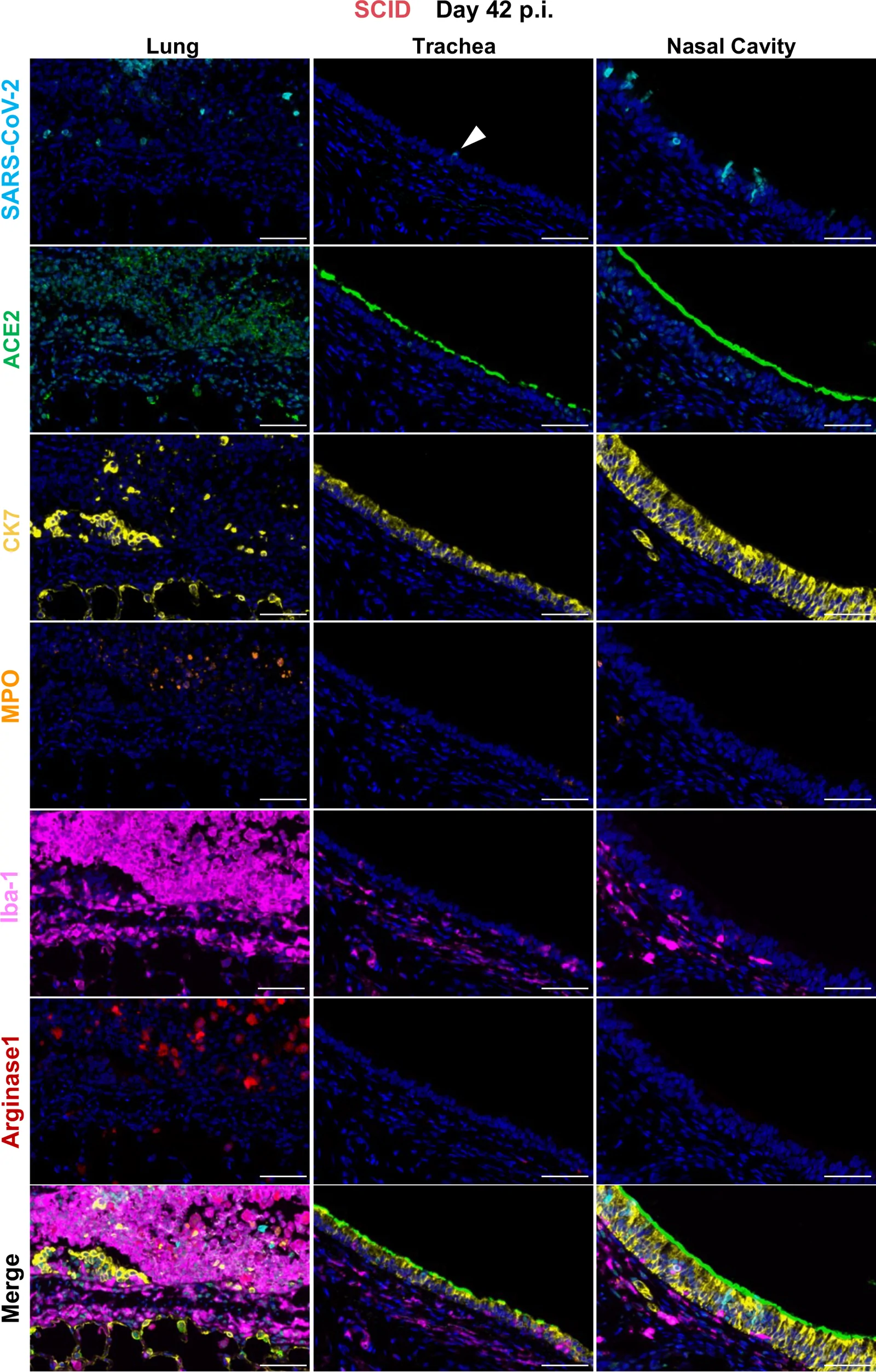
Multiplex immunofluorescence analysis of persistent epithelial infection and immune cell infiltration at 42 days post-infection. Representative multiplex immunofluorescence-stained images of lung, trachea, and nasal cavity tissue harvested from SCID rats at 42 dpi, showing SARS-CoV-2 antigen (cyan), ACE2 receptor (green), the epithelial cell marker CK7 (yellow), the neutrophil marker MPO (orange), the M2 macrophage marker arginase-1 (red), and the pan-macrophage marker Iba-1 (magenta). The bottom row shows merged images of all channels. DAPI nuclear staining (blue) is also shown. Scale bar = 50 µm.

On Day 42 p.i., persistent infection was observed in the lungs of SCID rats (Fig. 5; the tissue sample shown is the same as that shown in Fig. 3B). Clusters comprising viral antigen-positive cells, ACE2-positive CK7-positive cells, ACE2-positive macrophages, arginase-1-positive macrophages, and neutrophils were observed within the lumen of the pulmonary bronchioles. A small number of viral antigen-positive cells were observed in the epithelial layer of the trachea and nasal passages; however, only mild infiltration by Iba-1-positive macrophages was observed. Although persistent infection was established, these findings suggest that the inflammatory response in the nasal cavity and trachea is less pronounced than that in the lungs. To provide a baseline context for these tissue-dependent differences, we examined the nasal cavity, trachea, and lungs of uninfected SCID and DMEM-inoculated WT rats (Fig. S3). The distributions of MPO- and Iba-1-positive cells were qualitatively comparable between the two groups in all three respiratory tissues, and no obvious differences were observed in the myeloid cell populations assessed using these markers. In contrast, H&E staining revealed clear differences in the lymphoid tissue architecture. Lymphoid aggregates or follicle-like structures were observed in the nasal cavity and lungs of WT rats but were poorly developed or absent in SCID rats.

### Viral evolution under weak immune pressure

**Fig 6:**
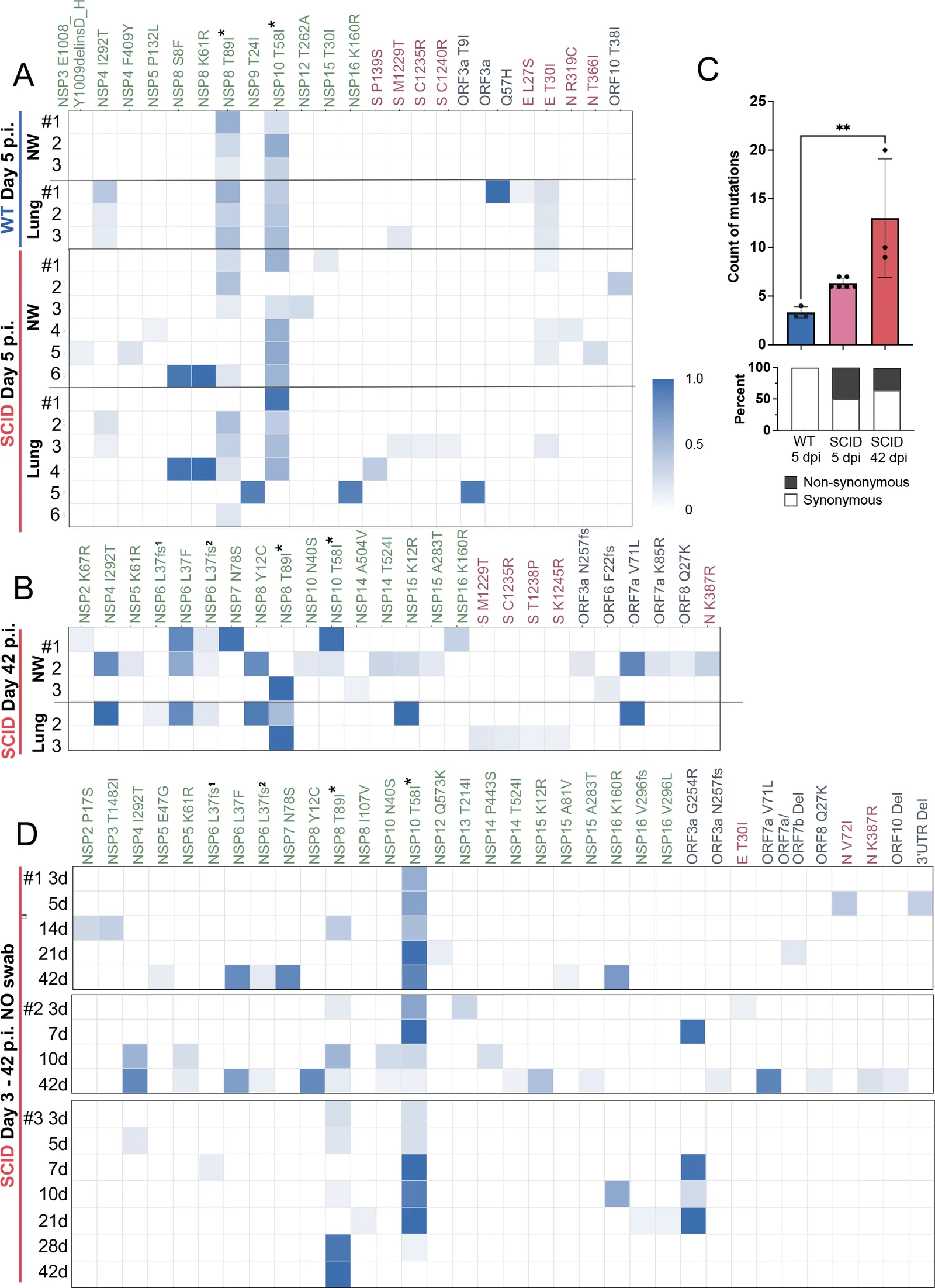
SARS-CoV-2 genomic mutations detected during persistent infection. (**A**) Comparison of mutation frequencies during early infection. Heatmaps showing the frequency of amino acid substitutions in SARS-CoV-2 genomes detected in nasal wash (NW) and lung samples from WT F344/Jcl (upper panels) and SCID (lower panels) rats at Day 5 p.i.. Color intensity indicates mutation frequency (0 [white] to 1.0 [dark blue]). (**B**) Mutation profile during persistent infection. Heatmaps showing the frequency of amino acid substitutions in SARS-CoV-2 genomes from NW and lung samples from SCID rats at Day 42 p.i. Color intensity indicates mutation frequency (0 [white] to 1.0 [dark blue]). (**C**) Analysis of mutation counts and types. Total mutation counts (upper panel) and the proportion of non-synonymous (black) and synonymous (white) mutations (lower panel) in WT (5 days p.i.) and SCID (5 and 42 days p.i.) rats. Each data point in the upper panel represents an individual rat. **P < 0.01; Kruskal–Wallis test followed by Dunn’s multiple comparisons test. (**D**) Temporal pattern of mutation accumulation. Accumulation of mutations in nasal/oral swabs (NO) from three individual SCID rats (#1-#3) from days 3 to 42 p.i.. Serial samples were obtained from the same rats. Color intensity indicates mutation frequency (0 [white] to 1.0 [dark blue]). Stars denote mutations that were detected initially as mixed variants in the original QHmusX inoculum (NSP8–T89I, 26.5%; NSP–10T58I, 46.3%). 1, an insertion at nucleotides 11,074–11,075 (– to T) ; 2, a deletion at nucleotide 11,083 (G to –) both resulted in an identical amino acid change.

To evaluate the viral evolution during persistent infection, we performed next-generation sequencing of viral RNA isolated from nasal washes and lung tissue homogenates. Comparison of viral genomes between days 5 and 42 p.i. revealed that SCID rats showed higher mutation counts over time than WT rats (Fig. 6A and B). Longitudinal analysis of viral RNA obtained from nasal/oral swabs revealed stepwise accumulation of mutations over the course of infection (Fig. 6C). The ratio of nonsynonymous to synonymous substitutions remained relatively constant throughout the observation period. Mutation mapping revealed preferential accumulation within genomic regions encoding non-structural proteins (NSPs) rather than in those encoding structural proteins typically targeted by adaptive immune responses (Fig. 6D). Individual SCID rats exhibited diverse mutation patterns, with some mutations detected transiently before reverting to the original sequence. Several substitutions recurred across animals.

### Transmissibility of SARS-CoV-2 in SCID rats harboring persistent infection

**Fig 7:**
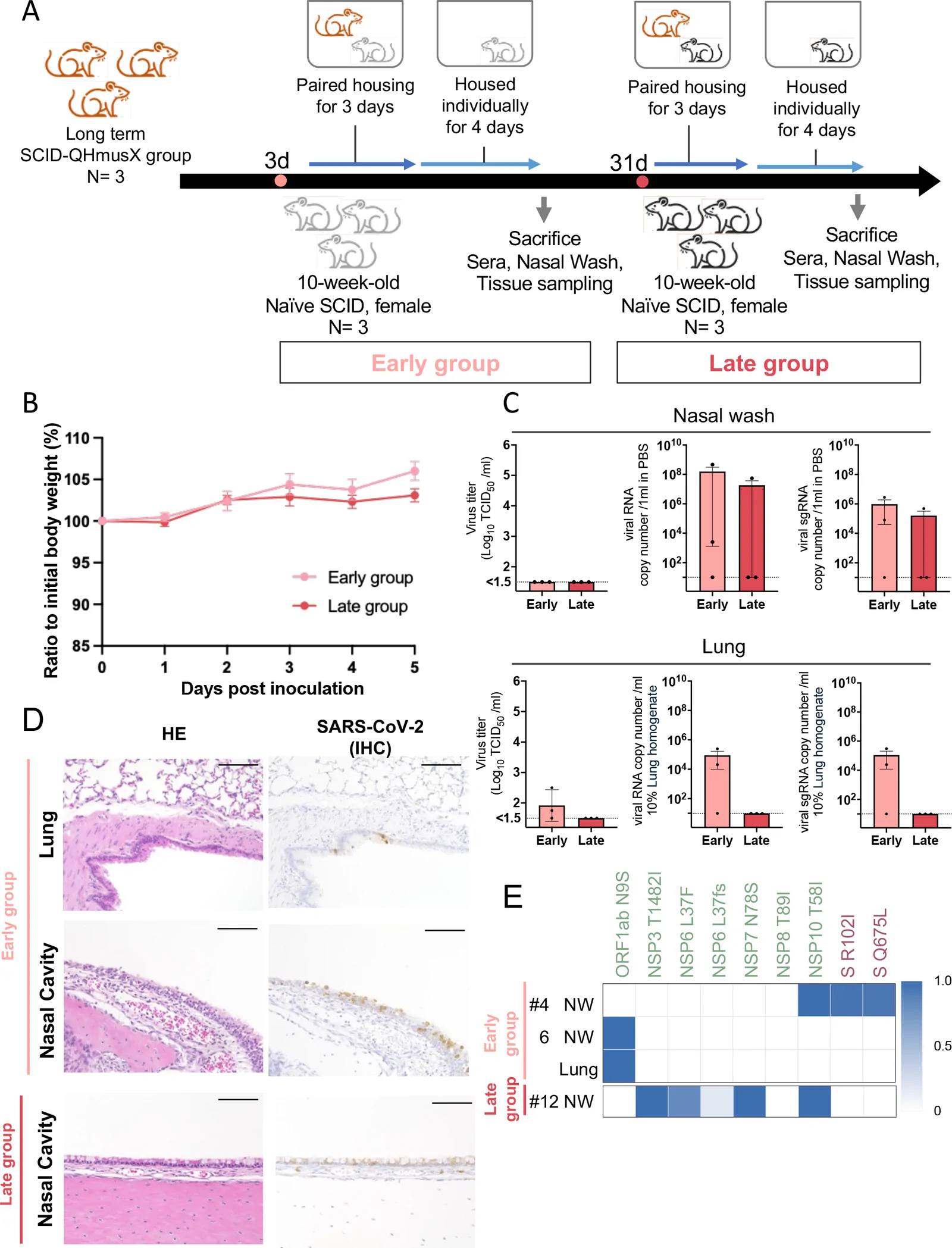
Transmission of SARS-CoV-2 from SCID rats to uninfected rats during the infection phase. (**A**) Experimental design. Uninfected (contact) SCID rats were co-housed with infected rats for 3 days, starting at 3 (early group) or 31 days (late group) p.i., followed by 4 days of individual housing before analysis. (**B**) Changes in body weight of contact rats. Body weight changes in the early and late groups were measured. The graphs show percentage changes in body weight relative to baseline plotted against days p.i. (**C**) Quantification of viral RNA in contact rats. Viral RNA copy number in nasal wash (upper) and lung homogenates (lower) from contact rats was measured. Data from early (light pink) and late groups (red) are shown. (**D**) Histopathological findings after transmission. H&E staining (left) and immunohistochemical staining (right) to detect SARS-CoV-2 antigen in contact rats after transmission. Images showing lung (upper panel) and nasal cavity (middle panel) tissues from the Early group and nasal cavity (lower panel) tissue from the Late group. Scale bar = 100 µm. (**E**) Mutation profile of transmitted viruses. Heatmaps showing the frequency of amino acid substitutions in transmitted SARS-CoV-2 genomes. Mutation patterns in nasal wash (NW) and lung samples from contact SCID rats in the early (upper panel) and late groups (lower panel) are shown. Color intensity indicates mutation frequency (0 [white] to 1.0 [dark blue]).

Finally, to evaluate whether individuals persistently infected with SARS-CoV-2 could transmit the infectious virus to other hosts, we conducted cohabitation experiments using rats that had been infected for 3 (early) or 31 days (persistent). Briefly, naïve SCID rats (*n* = 3) were cohoused with infected rats (*n* = 3) for 3 days, after which the contact rats were housed individually for 4 days before terminal sampling (Fig. 7A). The subsequent 4-day individual housing period was included to allow infection acquired during co-housing to be established before terminal sampling; the experiment was not designed to identify the precise timing or route of exposure. There were no significant differences between the early and persistent infection groups with respect to changes in body weight, suggesting a comparable clinical presentation, regardless of the timing of exposure (Fig. 7B). Viral RNA was detected in the nasal wash fluid and lung homogenates from all contact rats, indicating successful transmission; however, viral loads in rats exposed during the persistent infection phase (Day 31) were lower in the late-exposure group than in contact rats exposed during the early phase (Day 3; Fig. 7C). Histological analysis confirmed the presence of viral antigen-positive cells in the lungs and nasal cavity of contact rats exposed during the early phase of infection as well as in the nasal epithelium of contact rats exposed during the persistent phase (Fig. 7D). Genomic analysis of the transmitted viruses revealed distinct differences in the mutational profiles depending on the timing of transmission. In contact rats in the early infection group, the virus transmitted to one rat carried additional mutations relative to the source virus, whereas viruses transmitted during the persistent phase of infection largely retained the sequence of the corresponding source virus (Fig. 7E). In all cases, the transmitted viruses retained their infectivity and replication capacity in contact with SCID rats, demonstrating that the infectious virus was shed continuously, even during the persistent phase.

## DISCUSSION

Here, we present a rat model of SCID that captures selected features of prolonged SARS-CoV-2 infection under profoundly immunodeficient conditions, including persistent viral replication, intra-host viral sequence variation, and contact transmission. Following intranasal inoculation with the mouse-passaged QHmusX strain, infected rats supported prolonged viral replication for at least 42 days without overt clinical disease, paralleling reports of prolonged viral shedding in some immunocompromised patients (9, 16, 34–36). Thus, this model provides a platform for studying long-term virus–host interactions, immune evasion, and viral evolution.

A key observation in this study was the relatively limited local inflammatory cell infiltration in the nasal cavity and trachea of infected SCID rats compared to that in the lungs. To provide a baseline context for this finding, we qualitatively compared uninfected SCID rats with DMEM-inoculated WT rats and found no obvious differences in the distribution of MPO- and Iba-1-positive cells in the nasal cavity, trachea, or lungs. Consistent with these findings, a previous characterization of this *Il2rg/Rag2*-deficient SCID rat line showed marked reductions in circulating T, B, and NK cells, whereas peripheral blood monocyte and granulocyte counts were comparable to those of WT F344/Jcl rats (33). However, that study evaluated circulating leukocyte populations rather than tissue-resident myeloid cell distribution, and our additional baseline comparison was qualitative. Therefore, although the reduced inflammatory cell accumulation observed after infection is unlikely to be explained solely by an obvious baseline deficiency of the MPO-positive and Iba-1-positive myeloid cell populations examined here, the respective contributions of tissue-specific immune architecture, infection status, and the *Rag2/Il2rg*-deficiency genotype cannot be fully distinguished.

Viral antigens persisted in ACE2- and CK7-positive epithelial cells in the nasal cavity and trachea, indicating an association between persistent epithelial infection and relatively limited local inflammatory cell infiltration in these tissues under profoundly immunodeficient conditions in the SCID rat model. In immunocompetent hosts, adaptive immune responses contribute to viral clearance, even when local innate cell infiltration is limited (37). Accordingly, these findings raise the possibility that the nasal cavity and trachea represent tissue sites where persistent epithelial infection can be maintained under profoundly immunodeficient conditions in this model. However, additional studies using complementary animal models are required to determine the relative contributions of the local tissue architecture, epithelial susceptibility, and host immune status to viral persistence.

Slow turnover of the airway epithelium may further contribute to this phenomenon. In young adults, renewal of the tracheal epithelium occurs over 24–42 days, and basal cells rarely divide during homeostasis (37, 38), making natural replacement unlikely to eliminate infected cells. A similar limitation was reported for cultured human nasal epithelial cells in which SARS-CoV-2 persisted for ≥28 days post-infection despite a robust antiviral signature and ongoing basal-cell proliferation; this indicates that epithelial turnover alone is insufficient for effective clearance (39).

The tissue-specific immune architecture also shapes the outcomes. The lungs harbor resident alveolar macrophages that provide continuous surveillance and orchestrate early antiviral responses while simultaneously regulating inflammation (40, 41). In addition, WT rats possessed the bronchus-associated lymphoid tissue (BALT; Fig. S2A, black arrows), in which the resident immune cells respond rapidly to viral antigens. In contrast, the nasal cavity and trachea contain relatively few resident macrophages and rely largely on the recruitment of peripheral immune cells, such as neutrophils and CCR2-dependent monocyte-derived tracheal macrophages, for protection and epithelial repair (42, 43). The structural context also differs: the simple alveolar monolayer, together with dense resident macrophages, favors rapid immune activation and viral clearance, whereas the pseudostratified ciliated epithelium of the nasal cavity and trachea, combined with mucociliary barriers, can substantially limit access, especially in immunodeficient settings (44, 45). Consistent with this, we observed viral antigen-positive cells not only in the superficial layer of the nasal epithelium but also in the deeper layers, which is in line with reports of persistent viral antigens in the human nasal mucosa (46) and raises the possibility of cell-to-cell transmission in addition to canonical ACE2-dependent entry. Indeed, ACE2-independent intercellular routes and membrane fusion can mediate spread, potentially enabling immune evasion and promoting persistence (47–49). Taken together, the present observations are consistent with the possibility that the local tissue context, epithelial susceptibility, and profound immune defects of this model contribute to persistent infection. However, the relative contribution of each factor is yet to be determined, in particular, the upper airway epithelial reservoir, in which structural constraints, slowed mucociliary clearance, sparse baseline myeloid surveillance, and limited turnover intersect may sustain low-level replication and shedding in the absence of adaptive immunity (46).

Consistent with a predominantly respiratory reservoir, low-level genomic viral RNA was detected in the blood of several SCID rats at Day 5 p.i. and in one longitudinal sample at Day 14 p.i., but was not detected at Day 42 p.i. The absence of detectable subgenomic RNA in blood, in contrast to its detection in tissue specimens from SCID rats, is consistent with viral transcription and replication being predominantly localized to respiratory tissues rather than representing sustained systemic replication. Viral RNA and occasional antigen signals were intermittently detected in intestinal samples; however, the near absence of subgenomic RNA and the lack of histological evidence suggest that gastrointestinal detection reflects swallowed airway secretions rather than sustained intestinal replication. Together, these findings support the concept that prolonged infection in this model is maintained predominantly within localized respiratory tissue reservoirs rather than through sustained systemic viral replication.

Longitudinal sequencing of the viral genome revealed that SCID rats accumulated significantly more mutations over time than the WT controls. These mutations were located predominantly in NSP-coding regions, whereas relatively few changes were observed in structural proteins that are typically targeted by adaptive immune responses. This mutation pattern appears to be consistent with a process driven by genetic drift; however, the possibility that innate immune pressures also contribute to shaping these mutational patterns cannot be excluded. Notably, the recurrent mutations observed in this study (including NSP6 L37F, E T30I, ORF10 T38I, and NSP16 K160R) have been reported as major mutations in persistent infection models using SCID and T cell-deficient mice, as well as in immunodeficient patients (23, 24, 50). The recurrence of some substitutions across animals is compatible with nonrandom selection; however, functional adaptation cannot be inferred from sequence data alone. In contrast, the transient appearance and reversion of other mutations were consistent with stochastic viral evolution in the absence of strong adaptive immune selection. Despite the identical housing conditions, each SCID rat followed a unique evolutionary trajectory. Similar to previous reports in immunocompromised patients (6, 15, 34), our findings suggest that prolonged infection in an immunocompromised host can provide an individual reservoir in which sustained viral replication permits the accumulation and turnover of viral sequence variations. These findings underscore the complex interplay between genetic drift and low-level innate immune pressures in shaping viral evolution during persistent infection. Understanding this balance is essential to anticipating the emergence of new variants in immunocompromised populations.

Our cohabitation experiments demonstrated that the infectious virus remained transmissible, even during persistent infection, although the viral loads in contact animals were lower when exposure occurred at later stages (i.e., 31 days p.i.). Genomic analysis suggests that viruses transmitted at an early stage sometimes carry additional structural mutations, whereas those transmitted during the persistent phase retain the source sequence more closely. These observations imply that transmissibility is maintained throughout persistent infection, but the genetic features of the transmitted viral populations may vary depending on the stage of infection (4, 18, 34). Although we did not assess the pathogenicity of the transmitted viruses, these findings highlight the need to clarify whether such differences affect the transmission efficiency or clinical outcomes of immunocompromised hosts.

This model provides a useful platform for evaluating antiviral strategies designed to eliminate persistent viral reservoirs and reduce contact transmission. In particular, emerging approaches targeting essential viral replication proteins, including the targeted degradation of NSP12 (51), may be especially relevant for persistent infections, although such therapeutic interventions are beyond the scope of the present study. Such applications remain prospective because therapeutic interventions were not tested in this study.

This study had some limitations. Although both groups were derived from the F344 background, they were not littermate controls. Therefore, residual substrain- or colony-related differences could not be completely excluded. Moreover, the small cohort size and limited long-term replication constrain statistical power and generalizability. Inter-animal variability has been observed in several outcome measures, and larger, prespecified studies are required to quantify effect sizes and determine the extent to which this variability reflects biological heterogeneity rather than sampling variation. The transmission experiment did not include uninfected donor sentinel controls and was not designed to identify the precise timing or route of viral exposure.

The biological characteristics of the model should be considered when interpreting these findings. Rats with SCID lack adaptive immunity, and clinical immunosuppression varies widely in terms of severity and mechanisms. Species differences between rats and humans may affect clinical translatability. In addition, the use of the mouse-passaged QHmusX strain, which carries spike substitutions that enhance rodent ACE2 usage, may have influenced viral infectivity, tissue tropism, persistence, and contact transmission observed in the present study. Consequently, these findings cannot be generalized to all circulating SARS-CoV-2 variants without direct experimental validation. SARS-CoV-2 variants from the delta lineage onward have been reported to utilize murine ACE2 (52), providing an opportunity for future studies to determine whether similar patterns of persistence can be reproduced using naturally evolved variants with intrinsic murine ACE2 compatibility.

Finally, the tissue distribution of persistent infections differed from that reported in many immunocompromised patients. Although persistent viral replication in this model is largely confined to the nasal cavity and trachea, clinical reports have described persistent viral replication in both the upper and lower respiratory tracts. These differences may reflect species-specific factors, differences in the nature and severity of the underlying immune defects, or characteristics of the mouse-adapted challenge virus. Accordingly, this model should be regarded as capturing selected features of persistent SARS-CoV-2 infection under profoundly immunodeficient conditions, rather than the full spectrum of persistent infection observed in humans.

Future studies employing single-cell transcriptomics and spatial profiling are necessary to identify the determinants of tissue-specific viral persistence. Extending the observation period beyond 42 days and evaluating antiviral interventions will further define the natural history of persistent infection and inform the development of future therapeutic strategies.

In summary, we established a rat model of SCID that supports prolonged SARS-CoV-2 infection in profoundly immunodeficient conditions. Persistent infection in this model was associated with persistent epithelial infection in the nasal cavity and trachea, together with relatively limited local immune cell infiltration. This model captures selected features of persistent SARS-CoV-2 infection under profoundly immunodeficient conditions and provides a useful experimental platform for investigating the mechanisms of prolonged infection, viral evolution, and contact transmission. The model may also facilitate future studies aimed at understanding and ultimately eliminating persistent viral reservoirs in immunocompromised hosts.

## MATERIALS AND METHODS

### Viruses and cells

The SARS-CoV-2 strain (QHmusX) was generated by 10 serial passages of the B.1 lineage virus (hCoV-19/Japan/QH-329-037/2020) in BALB/c mice (31). VeroE6/TMPRSS2 cells (JCRB1819, obtained from the National Institute of Biomedical Innovation, Health and Nutrition, Osaka, Japan) were used for virus propagation and testing. The cells were maintained at 37°C/5% CO₂ in Dulbecco’s Modified Eagle Medium (DMEM, Sigma-Aldrich, low glucose) supplemented with 10% fetal bovine serum (FBS), 50 IU/mL penicillin G, and 50 μg/mL streptomycin (10 DMEM). Viral titers were measured using the TCID_50_ method in VeroE6/TMPRSS2 cells and calculated using the Behrens–Kärber method. All titers are expressed as TCID_50_/mL.

### Animal experiments

#### Animals

Severe combined immunodeficient (SCID) (F344-*Il2rg/Rag2^em1Iexas^*; NBRP Rat No. 0895, University of Tokyo) and WT F344/Jcl rats (CLEA, Japan) were used in this study. The SCID line was generated by CRISPR/Cas9-mediated disruption of the *Il2rg* and *Rag2* genes in an inbred F344 background. Age-matched female WT F344/Jcl rats were selected as controls because they share the same inbred F344 genetic background. The two groups were not used as littermate controls. SCID rats lack B and T cells and have markedly reduced NK cell function, although their growth and development are similar to those of WT rats. All rats were maintained under specific pathogen-free conditions, and 9-week-old female rats were used.

#### Infection experiment

Anesthetized rats were inoculated intranasally with 10^5^ TCID_50_/50 μL of QHmusX or with DMEM as control. The rats were assigned to short- (5 days p.i.) or long-term (42 days p.i.) groups. Body weights were monitored over time. Nasal and oral swabs, body surface swabs, blood, and fecal samples were collected at multiple time points. At the end of the experiment (day 5 p.i. or 42 p.i.), the rats were euthanized using an overdose of isoflurane anesthesia, and nasal washes, lung tissues, and various organs were collected.

#### Transmission experiments

To evaluate transmissibility, SCID rats in the early (Day 3 p.i.) and persistent infection phases (Day 31 p.i.) were paired with uninfected contact SCID rats (10 weeks old, *n* = 3 per group). Immediately before the co-housing period, one infected donor rat and one naïve contact rat were transferred to a newly prepared cage containing fresh bedding and water and co-housed for 3 days. Infected rats (#1–3) were paired with uninfected recipients as follows: in the early group (day 3 p.i.), I#1 was paired with U#6, I#2 with U#4, and I#3 with U#5; and in the late group (day 31 p.i.), I#1 with U#12, I#2 with U#10, and I#3 with U#11. After separation, the contact rats were housed individually for an additional 4 days before sample collection. Changes in body weight were recorded, and viral RNA loads in the nasal wash fluid and lung tissues were analyzed.

#### Respiratory function tests

Respiratory function parameters (Penh, enhanced pause parameter; Rpef, time-to-peak expiratory flow ratio) were measured using a whole-body plethysmography system (Buxco). Briefly, rats were placed in non-restrained plethysmography chambers and allowed to acclimate for 5 min, after which data were collected over a period of 10 min. Respiratory function was monitored repeatedly at days 0, 3, and 5 p.i. in the short-term group and at days 0, 3, 6, 9, 14, 28, and 42 p.i. in the long-term group to assess both the early and persistent phases of infection (Fig. 1C). Data were acquired and analyzed using FinePoint software, and changes in airway resistance and breathing patterns were evaluated.

### RNA extraction and detection of viral RNA

#### Sample collection and processing

Oral/nasal and body surface swabs were suspended in 500 μL of physiological saline solution. Nasal wash fluid was collected by washing the nasal cavity of euthanized rats with 1 mL of 0.1% BSA in phosphate-buffered saline (PBS) supplemented with penicillin G (20 IU/mL), streptomycin (20 μg/mL), and amphotericin B (1 μg/mL). Lung tissues were ground using a mortar and pestle in the presence of sterile quartz sand and diluted with DMEM to obtain 10% (w/v) homogenates. The lung homogenates were prepared in DMEM containing 2% FBS, penicillin G (50 IU/mL), and streptomycin (50 μg/mL) (2DMEM).

#### RNA extraction

RNA extraction was performed using Maxwell RSC instruments and corresponding kits (Promega). RNA was eluted in 50 μL of nuclease-free water. The extracted RNA was quantified using a NanoDrop spectrometer (Thermo Fisher Scientific).

#### Real-time RT-PCR

Real-time RT-PCR to detect and measure viral RNA was performed using the QuantiTect Probe RT-PCR Kit (Qiagen) and the following primers and probes targeting the SARS-CoV-2 N gene: NIID_2019-nCOV_N_F2: 5′-AAATTTTGGGGACCAGGAAC-3′; NIID_2019-nCOV_N_R2:5′-TGGCAGCTGTGTAGGTCAAC-3′; and NIID_2019-nCOV_N_P2: 5′-FAM-ATGTCGCGCATTGGCATGGA-BHQ-3′.

Subgenomic RNA was detected using the following set: SARS2-LeaderF60: 5′-CGATCTCTTGTAGATCTGTTCTCT-3′; SARS2-N28354R: 5′-TCTGAGGGTCCACCAAACGT-3′; and SARS2-N28313Fam: 5′-FAM-TCAGCGAAATGCACCCCGCA-TAMRA-3′.

The reaction conditions were as follows: reverse transcription at 50°C for 30 min, followed by initial denaturation at 95°C for 15 min, 40 cycles of denaturation at 94°C for 15 s, and annealing/extension at 60°C for 60 s. Analysis was performed using a Light Cycler 480 (Roche). Five microliters of the RNA eluate was used for each RT-qPCR reaction, and viral RNA copy numbers for swab samples were expressed as viral RNA copies per swab.

### Neutralizing antibody titer measurement

Neutralizing antibody titers were measured using methods described previously (31). Each serum sample was heat-inactivated at 56°C for 30 min, then incubated with SARS-CoV-2 (100 TCID_50_/well) and inoculated into VeroE6/TMPRSS2 cells. Neutralization activity was assessed by monitoring the inhibition of cytopathic effects (CPE), and the highest serum dilution that completely prevented CPE was defined as the neutralizing antibody titer (NT titer).

### Histopathological analysis

#### Preparation of tissue specimens

Lung and other organ tissues were fixed in 10% phosphate-buffered formalin, embedded in paraffin, and sectioned (thickness, 4–5 μm). The nasal cavity tissue was decalcified in 10% EDTA buffer (pH 7.4, Dojindo) for 48 h. Sections were mounted on slides, stained with hematoxylin and eosin (H&E), and evaluated under a light microscope (Olympus; 100–400×) or a BZ-X810 microscope (Keyence, 40–200×).

#### Baseline histopathological comparison

To provide baseline histopathological data, the nasal cavity, tracheal, and lung tissues from uninfected SCID and DMEM-inoculated WT rats were examined. Sections were evaluated by H&E staining and immunostaining for MPO and Iba-1. Representative sections from each tissue were compared qualitatively to assess the baseline distribution of MPO- and Iba-1-positive cells and the organization of lymphoid tissue structures. Quantitative cell counting was not performed for this baseline comparison.

#### Immunohistochemical staining

Formalin-fixed, paraffin-embedded tissue sections were subjected to antigen retrieval by autoclaving in a retrieval solution (pH 6.0, 121°C, 10 min; Nichirei). SARS-CoV-2 antigens were detected using a polymer-based method (Nichirei-Histofine Simple Stain Rat MAX PO (R); Nichirei Biosciences) with an in-house rabbit anti-SARS-CoV-2 N antibody as the primary antibody. Diaminobenzidine was used for color development, and hematoxylin was applied for 10 s as a counterstain.

#### Multiplex immunofluorescence staining

Tissue sections were processed using an automated stainer (Leica Bond RXm; Leica Biosystems). A multiplex immunofluorescence assay was performed in a stepwise manner. First, each primary antibody was evaluated using single-marker DAB staining to determine the optimal antibody dilution and antigen retrieval conditions. Antibody–opal fluorophore pairings were selected according to antigen abundance, staining intensity, and spectral compatibility. For each primary antibody, the temperature, duration, and buffer pH of the heat-mediated stripping step performed after antibody detection were evaluated to determine the appropriate stripping conditions. Finally, the multiplex staining patterns were compared with those of the corresponding single-marker stains to confirm that the expected staining specificity and cellular localization were preserved. Based on these optimization results, the final antibody staining order and antibody-opal fluorophore pairings were established (Ref: https://www.akoyabio.com/wp-content/uploads/2021/12/Akoya_Opal_Assay_Development_Guide.pdf). Details of the antibodies, staining order, antigen retrieval, stripping conditions, and Opal fluorophore assignments are summarized in Table S1. Sequential multiplex staining was performed using heat-mediated antibody stripping between each staining cycle. Nuclei were counterstained with DAPI. Slides were scanned using a Vectra Polaris fluorescence slide scanner (Akoya Biosciences) and analyzed using inForm software (Akoya Biosciences).

#### Spatial analysis of immun*e* and virus-infected cells

To investigate the differences in tissue-specific immune responses between WT and SCID rats during the early phase of SARS-CoV-2 infection, a detailed spatial analysis was performed using respiratory tissues collected on Day 5 p.i. The spatial distribution of M2 macrophages (arginase-1-positive) and neutrophils (MPO-positive) relative to the viral antigen-positive regions was assessed using the multiplex immunofluorescence staining method described above. Images captured by Vectra Polaris (Akoya Biosciences) were analyzed using inForm software (Akoya Biosciences) to provide quantitative measurements of the distance between infected and immune cells, the number of infected cells, and the ratio of immune cells to infected cells.

### Next-generation sequencing analysis

Viral RNA extracted from nasal/oral swabs, nasal washes, and lung homogenates was used for full-length analysis of the viral genome. Methods and analyses were performed as previously described (53). Amplification of the complete SARS-CoV-2 genome was performed using a modified method based on the ARTIC Network protocol, with some primers replaced or added (54–56). Viral cDNA synthesis was performed using the Q5 Hot Start DNA Polymerase (New England BioLabs) and the Luna Script RT Super Mix Kit (New England BioLabs), followed by multiplex PCR using ARTIC-N5 primers (used in two separate primer pools). These DNA fragments were prepared as libraries using the QIAseq FX DNA Library Kit (Qiagen) and sequenced on an iSeq 100 and MiSeq (Illumina). To generate consensus sequences, the obtained reads were mapped to the SARS-CoV-2 reference genome (MN908947.3) using the CLC Genomics Workbench (version 22; Qiagen). The frequency of mutations at each amino acid position was also calculated. Sequence data were deposited in the DNA Data Bank of the Japan (DDBJ) Sequence Read Archive (BioProject accession numbers listed).

### Statistical analysis

Statistical analyses were performed using GraphPad Prism 9. Given the small sample size (*n* = 3 per group), the analyses were primarily descriptive. Where applicable, differences between groups were assessed using one-way analysis of variance (ANOVA) or the Mann–Whitney *U* test; however, the results should be interpreted as indicative trends rather than conclusive statistical differences.

## Data Availability

All BioSample records generated in this study have been deposited in the DDBJ BioSample database under accession number PRJDB23534, with the associated run accession numbers DRR719436–DRR719478. The full genome sequence of the mouse-passaged SARS-CoV-2 QHmusX strain used in this study has been deposited in the DNA Data Bank of Japan under accession no. LC605054. SCID rats (F344-Il2rg/Rag2em1Iexas; NBRP Rat No. 0895) were used in the NBRP Rat Project (https://ut-rat.jp). All other study data are included in the article and/or Fig. S1.

## SUPPLEMENTAL MATERIAL

Supplemental table and figures spectrum.04105-25-s0001.pdf Table S1, Fig. S1 to S3.

ASM does not own the copyrights to Supplemental Material that may be linked to, or accessed through, an article. The authors have granted ASM a non-exclusive, world-wide license to publish the Supplemental Material files. Please contact the corresponding author directly for reuse.
