# Supplementary material for: Persistent SARS-CoV-2 infection in SCID rats reflects immune vulnerability of the upper airway and trachea
Source: Microbiol Spectr. 2026 Oct 1;14(11):e04105-25. doi: 10.1128/spectrum.04105-25 (PMC13729136; doi:10.1128/spectrum.04105-25)
Supplement: Supplementary file 1 [file spectrum.04105-25-s0001.pdf]

S1 Table Optimized antibody panel and staining conditions for multiplex immunofluorescence

Order	Marker	Supplier	Catalog#	Dilution	Antigen retrieval /Stripping condition	Opal fluorophore
1	CK7	Abcam	AB181598	1:4000	pH9, 95℃, 20 min	Opal 570
2	Arginase-1	Cell Signaling Technology	93668S	1:200	pH6, 95℃, 20 min	Opal 690
3	ACE2	Thermo Fisher Scientific	MA5-32307	1:100	pH6, 95℃, 20 min	Opal 520
4	MPO	Proteintech	22225-1-AP	1:800	pH9, 95℃, 20 min	Opal 620
5	SARS-CoV-2	In-house	In-house	1:2000	pH6, 95℃, 20 min	Opal 480
6	Iba-1	FUJIFILM Wako	019-19741	1:1600	pH6, 95℃, 20 min	Opal 780

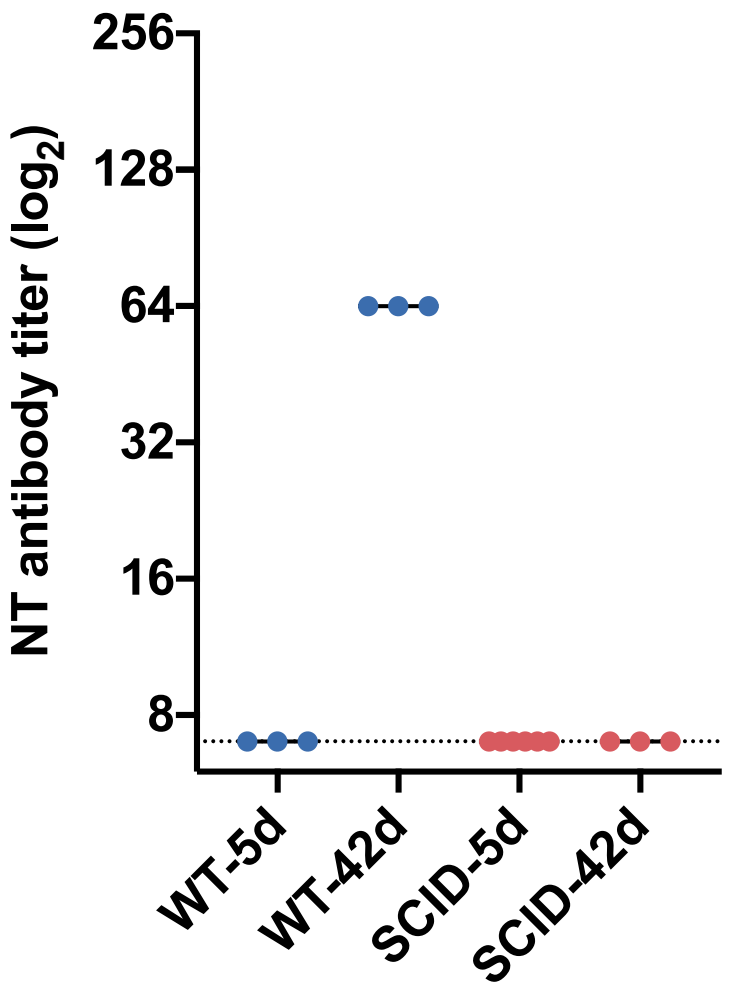

S1 Fig. Immune responses and viral replication dynamics. Temporal changes in neutralizing antibody titers. Neutralizing antibody titers ($\log_2$) in WT F344/Jcl (blue) and SCID (red) rats over time post-infection. Each point represents individual animal data.

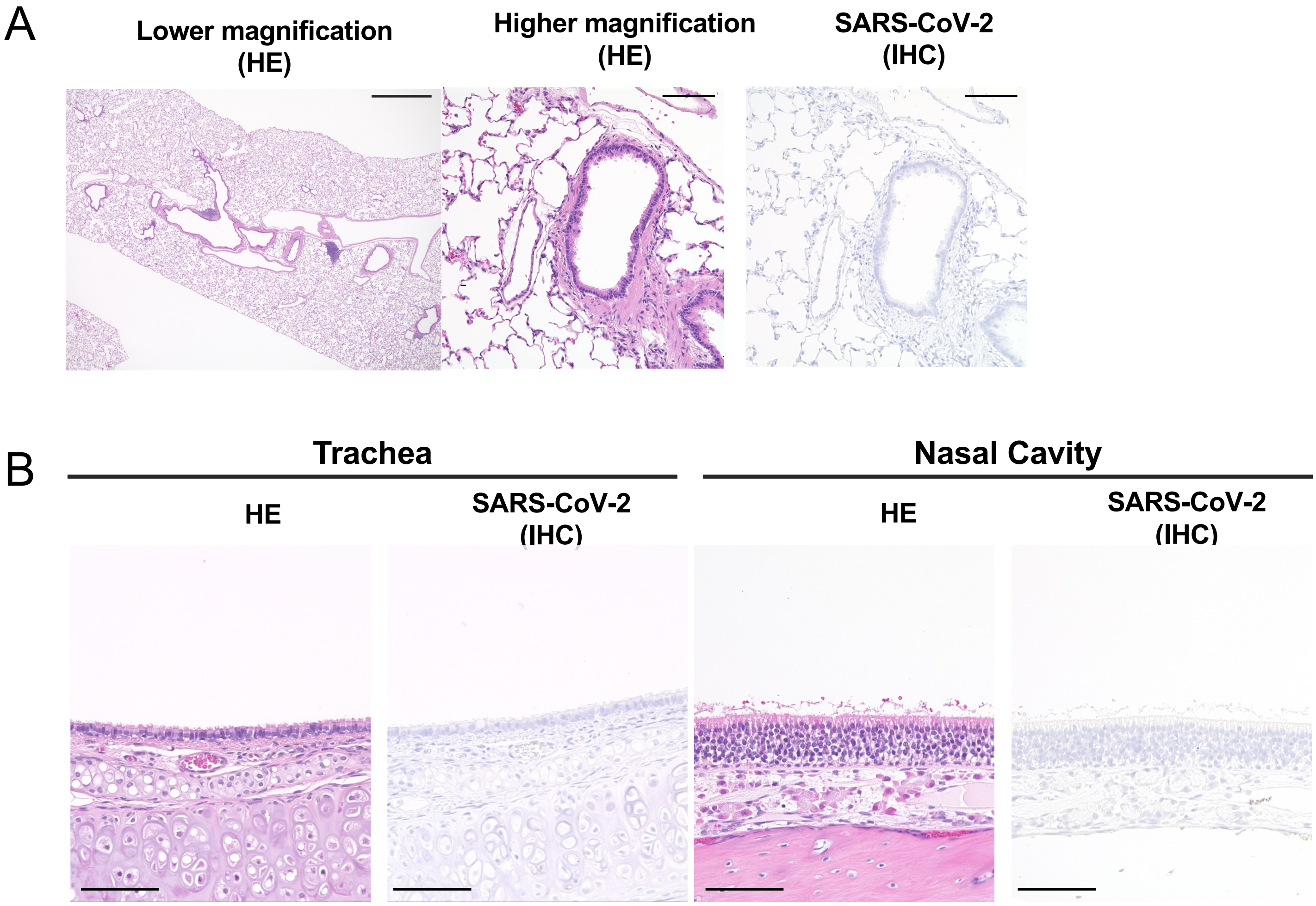

S2 Fig. Histopathological analysis of WT F344/Jcl rats at 42 days post-infection. (A) Lung tissue pathology. Representative images of lung tissues from WT F344/Jcl rats at 42 days post-infection stained with H&E (left and center) and SARS-CoV-2 immunohistochemistry (right). Scale bar = 100 µm. (B) Upper respiratory tract pathology. Corresponding trachea (upper panels) and nasal cavity (lower panels) sections stained with H&E (left) and SARS-CoV-2 antigen immunohistochemistry (right). Scale bars = 100 µm.

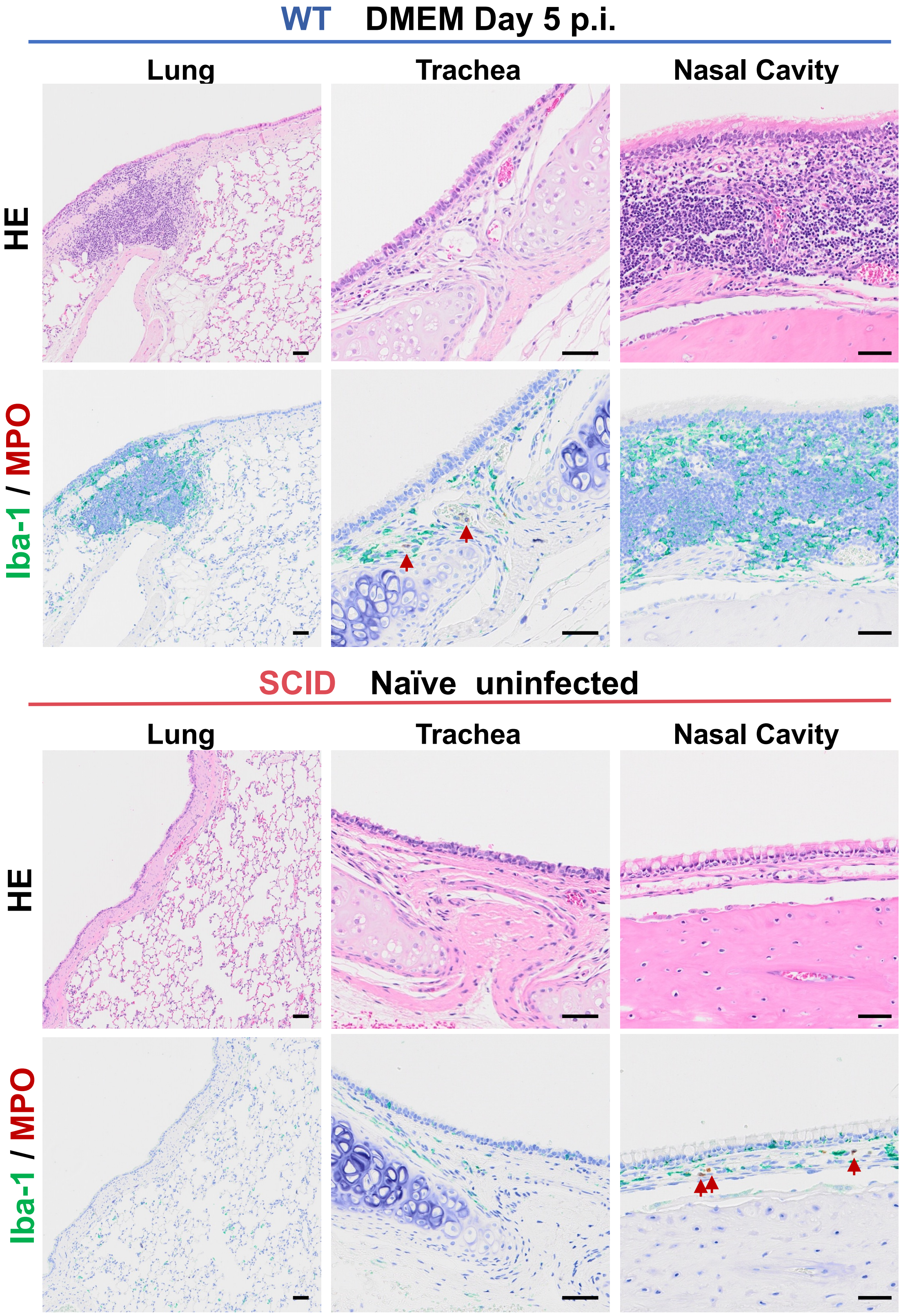

S3 Fig. Histopathological and immunohistochemical comparison of respiratory tissues from DMEM-inoculated WT F344/Jcl rats and naïve SCID rats. Representative H&E-stained and double immunohistochemical images of the lung, trachea, and nasal cavity from WT rats at 5 days after DMEM inoculation and 11-week-old naïve, uninfected SCID rats. Iba-1-positive cells are shown in green and MPO-positive cells in brown (arrows). Nuclei were counterstained with hematoxylin. Scale bars = 50 μm.
